# Current News and Opinion

**Published:** 1886-10

**Authors:** 


### Current News and Opinion.

### MEMORIZING DOSES.

Dr. G. A. Wiggins, of Philadelphia (*Med. World*, August, 1886), gives some general rules, with their exceptions, which are thoroughly reliable :

I. The dose of all infusions is 1 to 2 ozs., except infusion of digitalis, which is 2 to 4 drs.

II. Dose of all poisonous tinctures is 5 to 20 minims, except tincture of aconite, which is 1 to 5.

III. Dose of all wines is from 1-2 to 1 fl. dr., except wine of opium, which is 5 to 15 minims.

IV. Of all poisonous solid extracts you can give 1-2 gr., except extract of calabar bean, which is 1-16 to 1-4 gr.

V. Dose of all dilute acids is from 5 to 20 minims, exceps dilute hydrocyanic acid, which is 2 to 8 minims.

VI. Dose of all aquæ is from 1 to 2 ozs., except aqua laurocerasus and aqua ammonia, which are 10 to 30 minims.

VII. Of all syrups you can give 1 drachm.

VIII. Dose of all mixtures is from 1-2 to 1 fl. oz.

IX. Dose of all spirits is from 1-2 to 1 fl. dr.

X. Dose of all essential oils is from 1 to 5 minims.

—*Medical and Surgical Reporter.*

### NEW ENGLAND DENTAL SOCIETY.

The twenty-fourth annual meeting of the New England Dental Society will be held at the office of the S. S. White Dental Manufacturing Co., No. 160 Tremont Street, Boston, Mass., on Thursday and Friday, Oct. 7th and 8th, beginning on the first day at 10 o'clock, A. M.

The annual address will be by Dr. Norman W. Kingsley, of New York.

Some of the papers and essays to be read at the meeting are :

Our Patients as We Find Them. By Dr. George O. Tuck, Gloucester, Mass.

Christian Science, or Metaphysical Treatment in Dentistry. By Dr. Edward N. Harris, Boston.

Essay. By Dr. Edward S. Niles, Boston.

Reminiscences and Observations. By Dr. Sullivan L. Ward, Lowell, Mass.

The International Medical Congress and the relation of the Dental Profession thereto. By Dr. A. M. Dudley, Salem, Mass.

Subjects for discussion will be :

Successes and Failures in the use of Local Anæsthetics.

Simple Methods for Regulating Teeth.

Treatment of Chronic Alveolar Abscess.

All respectable members of the profession are invited to attend. An effort will be made to secure free return tickets over all railroads.

A. M. DUDLEY, Sec'y.

---

### "A DENTIST SHOULD HAVE CLEAN, WHITE HANDS."

The following hints will be found of service in accomplishing the desired end : A little ammonia or borax added to the water you wash your hands with, and that water just lukewarm, will keep the skin clean and soft. A little oatmeal mixed with the water will whiten the hands. Many people use glycerine on their hands when they go to bed, wearing gloves to keep the bedding clean; but glycerine does not agree with every one. It makes the skin hard and red. These people should rub their hands with dry outmeal and wear gloves in bed. The best preparation for the hands at night is white of egg with a grain of alum dissolved in it. White of egg, barley flour and honey, is a good application, but not better than oatmeal. The roughest and hardest hands can be made soft and white in a month's time by doctoring them a little at bed-time, and all that is required is a nail-brush, a bottle of ammonia, a box of powdered borax, and a little fine white sand to rub the stains off, or a cut of lemon, which will do even better, for the acid of the lemon will clean anything.

---

### INCISOR TOOTH IN ORBIT.

Dr. Ward Cousins recently showed to an English Medical Society an incisor tooth removed from the orbit of a child two years of age. It was perfect in outline and structure. Mr. Tracy did not regard it as the product of a dentigerous cyst, but as a specimen of a displaced tooth during an early stage of development.

### THE AMERICAN DENTAL SOCIETY OF EUROPE.

The meeting for 1886 was very well attended, and some good papers were read, which will appear in due time in this journal.

Dr. Miller was, by unanimous vote, authorized to draw upon the treasurer for all available funds (which amounts to about 1200 m.), for the expenses of his experiments in physiology and pathology.

This society believes in putting its money where it will do the most good. It will be remembered that three years ago it paid 800 m. for a microscope to be used by its members who are microscopists, and two years ago it sent 400 m. to the widow of Marshall H. Webb. The members assess themselves freely, and then each year use all the money for promoting scientific investigation. Our American professional brethren in Europe seem to be making quite as good progress as their *confreres* at home.

The following is the list of officers elected for the ensuing year:

*President*—Dr. E. P. George.

*Vice-President*—Dr. C. T. Terry.

*Secretary*—Dr. E. A. Galbraith.

*Treasurer*—Dr. Wm. Sachs.

---

### MINNESOTA.

The third annual meeting of the Minnesota State Dental Association was held at the State Capitol, St. Paul, commencing July 21st and continuing three days. The next meeting will be in Minneapolis, the second Wednesday in July, 1887.

The officers for the ensuing year are:

*President*—Dr. C. M. Bailey, Minneapolis.

*Vice-President*—Dr. H. L. Cruttenden, Northfield.

*Recording Secretary*—Dr. D. W. Edwards, Le Seur.

*Corresponding Secretary*—Dr. L. C. Gould, St. Paul.

*Treasurer*—Dr. Reid, Minneapolis.

M. G. Jenison, Sec'y.

---

### TO DENTISTS.

The undersigned is desirous of obtaining, at as early a day as possible, all Bills, Charters, Statutes, Statistical Records, Books, Pamphlets or Documents, relating directly or indirectly to Dental Education, Examination and Qualification. The receipt of any of the above will be acknowledged with thanks, and postage and other expenses paid.

Frank Abbott, M. D.,
22 West 40th Street, New York.

---

### OHIO STATE DENTAL SOCIETY.

The second annual meeting of the Ohio State Dental Society (reorganized) will be held in Toledo, Ohio, beginning on Tuesday, Oct. 26th, at 10 A. M.

J. R. Callahan, Sec.

### UNION MEETING. FIFTH, SIXTH, SEVENTH AND EIGHTH DISTRICT DENTAL SOCIETIES OF NEW YORK.

There will be held in Rochester, N. Y., on Oct. 26th and 27th, a joint convention of the Fifth, Sixth, Seventh and Eighth District Dental Societies of the State of New York. This includes one-half the districts of the State. Prominent members of the profession will be present; clinics will be given, and papers on practical subjects will be read and discussed. Members of the profession are invited to be present. Those who attend will improve a rare opportunity, while those who stay away will have much to regret. Note the date in your appointment book, and be prepared to come.

CHAS. T. HOWARD, Rec. Sec'y 7th District.

---

### FIFTH DISTRICT DENTAL SOCIETY.

The Fifth District Dental Society will hold its eighteenth semi-annual meeting at Syracuse, Monday, Oct. 25, 1886. The sessions will be held at the Vanderbilt House. Visiting dentists will be cordially welcomed.

C. J. PETERS, Rec. Sec.

---

THE MUMMY OF RAMESES II, of Egypt, the Sesostris of the Greeks, and the Pharaoh of the Bible, under whose reign the exodus of the Jews occurred, has lately been unrolled at Boulac, a suburb of Cairo, in Egypt, in the presence of the Khedive, the director general of the excavations and antiquities in Egypt, and other high dignitaries and learned men from all countries. It was discovered in the subterraneous hiding place at Dayrel-Bahari, and its identity was fully established by the ancient inscriptions.

Rameses ruled in Egypt more than 3,200 years ago. His reign was a very long one—67 years—and he must have been nearly 100 years of age at the time of his death. He was an exceedingly warlike potentate, and conquered a number of countries. He also built some of the most enduring of the wonderful Egyptian monuments of skill and labor, and ranks as one of the greatest monarchs who ever sat upon the throne.

The head was found to be long, and small in proportion to the body. The top of the skull was quite bare. On the temples there were a few sparse hairs, but at the poll the hair was quite thick, forming smooth, straight locks, about five centimetres in length. White at the time of death, they had been dyed a light yellow by the spices used in embalming. The forehead was low and narrow, the superciliary ridge prominent, the eyebrows thick and white, the eyes small and close together, the nose long, thin, and hooked. The temples were sunken, the cheek bones very prominent, the ears round, standing far out from the head and pierced for the receiving of earrings. The jawbones were massive and strong, and the chin prominent, the mouth small but thick-lipped, and filled with some kind of a black paste. On clearing this away a set of white and well-preserved teeth came in view, which were much worn and very brittle. The moustache and beard were thin, and seem to have been kept shorn during life.

How Many of the readers of this journal have tried the different preparations of Parke, Davis & Co., advertised upon the fourth cover page. This firm makes a specialty of preparations for dental use, and dentists should appreciate this fact. Their mark upon any package is a sufficient guarantee of its purity, for there is no firm which has a higher standing in the trade, and none is more enterprising in presenting the new drugs. They send with their preparations property and dose lists, and are always ready courteously to answer letters of inquiry. There should be a better knowledge of drugs among dentists, and it cannot be better obtained than through that reliable firm, Parke, Davis & Co. Write to them, and mention this journal.

---

A Gentleman received a note from his lawyer which he was unable to decipher. On his way to his office he met a friend at the door of a drug store. The friend, after vainly attempting to read the note, suggested that they step in and hand it to the druggist without comment. The druggist, after studying it in silence for a few minutes, stepped behind his prescription case, and in a short time returned with a bottle of medicine, duly labeled and bearing directions. When the gentleman saw his lawyer, he was informed that the note was a notice for him to call at his office between three and four P. M., of the following day. It is a pretty difficult matter to "stick" the regulation druggist.

—*Medical Age.*

---

Dr. Geo. W. Melotte, of Ithaca, N. Y., has devised a set of soldering clamps which will be found very useful, especially to those who are engaged in making crown and bridge-work. Into a wooden handle are inserted iron fingers, the ends of which are adapted to hold securely different forms while soldering. They are made of iron, so that they may be heated and cooled repeatedly, or plunged into water while hot, without hardening. They will frequently obviate investment for the purpose of holding parts together, and will save the time necessary in making special clamps, or in tying together with binding wire, besides holding small pieces more securely.

---

William Wood & Co., medical publishers, announce that they will not hereafter pay transportation charges on copies of their books intended for editorial notice. That is, in addition to about twenty-five dollars' worth of advertising for a three dollar book, they now ask the editor or journal to assist in paying a part of the legitimate expenses of running their business. And yet it is probable that there will be editors so lacking in dignity as to accept of books at their hands. It will pay to keep an eye on the "book notices" of some medical journals. As for William Wood & Co., it always was a good firm to avoid dealing with.

---

The Director of the American mint reports the amount of gold and silver used in the arts and manufactures in the United States for 1883, to be: Gold, about $15,000,000; silver, about $5,555,500, making a total of $20,555,500. There was consumed for dental supplies: Gold, about $39,900; silver, $6,735, making a total of $44,635.

A SCANDINAVIAN CUSTOMER was having a liniment prepared by a drug clerk, and calling for the ingredients by the five and ten cents' worth. After it was supposed to be finished, corked and delivered, he shook it up, looked at it, and said, "I will have five cents' worth of *nail olea* put in." Clerk was dubious, and the Norwegian explained, "I don't know what you call it in English; it is oil of big nail." "Oil of big nail, oh! is it oil of spike?" and he answered, "Oh yes, that's it, oil of spike."—*Medical and Surgical Reporter.*

---

A HYPERCRITICAL MEDICAL MAN found fault with Mary Anderson because of her assuming the stiffness of a corpse immediately on being killed in one of her plays, holding that cadaveric rigidity does not set in until five or six hours after death The fair actress naively inquired whether it would not be a very severe strain on the patience of the audience to oblige it to wait for six hours until she became stiff after the fashion of nature.

---

LODGER TO CHAMBERMAID, "Mary, this is the stillest house I was ever in. The landlord and his wife must live like angels in Heaven together; I haven't heard one single sound since I've been here."

MARY, "That's all very nice just now, Mr. Blank, but wait till they make friends again. They quarreled a fortnight ago and haven't spoken to each other since."

---

THE MICROSCOPE, a journal formerly owned and published by Prof. Stowell, has been purchased and will be edited by the following Detroit gentlemen, well known as workers in the field of microscopy, viz: Drs. W. P. Munton, Geo. Duffield, Frank Brown, and C. G. Jennings. Unquestionably they will increase the good work in this field of journalism, so well begun by Professor Stowell.

---

THE FOLLOWING are the component parts of a cigar: Acetic, formic, butyric, valeric, prussic, and proprionic acids; also creosote, ammonia, sulphuretted hydrogen, pyridine, picoline, and rubidene, to say nothing of the cabbagine and burdockic acid. That's why you can't get a good one for less than five cents.

—*Medical Age.*

---

THE MEDICAL RECORD modifies some of the ancient adages to suit more modern tastes and advanced ideas, as follows: A cut in time saves nine. Ambulances alter cases. What can't be cured must be regularly treated at the regular rates.

---

A PLEDGE OF LOVE, a veritable cupid, has made its appearance in the family of Dr. I. N. Love, editor of *The Weekly Medical Review.* Weight 13 lbs. A substantial token of Love, indeed. Congratulations.

---

PASTEUR has hitherto been unable to practice medicine, or any specialty in it, as he has never received a medical degree. That is now obviated, as an honorary degree has been conferred upon him.

---

BUFFALO boasts of being the only city in New York in which the births are in excess of the deaths.

